# GDT-SwinKid: A hybrid model for precise renal lesion analysis

**DOI:** 10.1371/journal.pone.0349285

**Published:** 2026-05-20

**Authors:** Thirupathi Rao N, V V Ramana CH, Eatedal Alabdulkreem, Ayman Aljarbouh, Samih M. Mostafa

**Affiliations:** 1 Department of Computer Science and Engineering, Vignan’s Institute of Information Technology (A), Visakhapatnam, Andhra Pradesh, India; 2 Vignan’s Institute of Information Technology (A), Besides VSEZ, Vadlapudi Duvvada, Visakhapatnam, Andhra Pradesh, India; 3 Department of Computer Sciences, College of Computer and Information Sciences, Princess Nourah bint Abdulrahman University, Riyadh, Saudi Arabia; 4 Department of Software Engineering, College of Computer Engineering and Science, Prince Mohammad Bin Fahd University, Al Khobar, Kingdom of Saudi Arabia; 5 Computer Science Department, Faculty of Computers and Information, Qena University, Qena, Egypt; University of Salerno: Universita degli Studi di Salerno, ITALY

## Abstract

Detecting and delineating renal lesions accurately remains a significant clinical problem due to the variety of kidney pathology and subtle differences in CT image interpretation. In this paper, we present the design of a next-generation hybrid model called GDT-SwinKid (Gamma Distribution-based Swin Transformer for Renal Lesions), which integrates the hierarchical feature attention mechanisms of Swin Transforms with a modified U-Net decoder and employs advanced statistical modeling (specifically through an adaptive Gamma distribution). The design of GDT-SwinKid allows for both precise extraction of fine details regarding kidney lesions, as well as achieving overall contextual awareness using cross-attention and Gamma-modulated feature refinement to address the drawbacks of existing approaches. Through extensive validation utilizing a large set of clinical datasets, GDT-SwinKid achieved better performance through segmentation and classification, obtaining Dice coefficients as high as 0.95, with AUC values approaching 0.99, when compared to leading transformers and convolutional models. An absolute improvement of 5–9% in Dice coefficient compared to conventional U-Net and Swin Transformer baselines, and an increase in AUC-ROC values approaching 0.99, outperforming existing hybrid and transformer-based methods on the same CT kidney dataset. The inclusion of explainable attention maps and deep supervision provides increased trust and accountability while enabling the rapid and robust integration of GDT-SwinKid into diagnostic pipelines for kidney imaging. GDT-SwinKid combines statistical sensitivity, hierarchical attention and clinical transparency to provide a new standard for automated kidney lesion analysis and to increase the reliability and use of newly developed AI techniques in renal imaging.

## 1. Introduction

Renal lesions consistently create challenges to clinicians, where effective intervention and patient management rely on accurate detection and diagnosis of renal lesions [1,2]. While technological advances in imaging provide increased diagnostic capabilities, distinguishing small or atypical renal findings continues to be problematic in most cases due to presenting findings that obscure the renal area and due to the presence of radiological backgrounds that may be confusing for the radiologist [3,4]. Recently, the application of deep learning models, including convolutional neural networks (CNN) and transformer-based architectures, is beginning to improve and modernize the field of medical image analysis, providing increased sensitivity to patterns not previously available. Even with considerable success, development of many traditional methods does not hold up well against the inherent statistical variability and intensity variability of kidney tissue imaging. Limitations in context awareness and overlooking small clinically relevant areas have motivated exploration of hybrid approaches that combine local feature extraction with global context modeling [5]. The developments of attention-based transformer methods, as well as statistical methods using Gamma distributions, are encouraging steps in this direction. However, development of robust, interpretive amalgamation of these methods for clinical needs is still an open question.

The current research presents GDT-SwinKid a hybrid paradigm developed to correctly characterize renal lesions based on a combination of modified U-Net segmentation decoder, Swin Transformer attention mechanisms, and Gamma-guided statistical modeling. GDT-SwinKid was developed in response to well documented gaps in sensitivity, interpretability, and lesion discrimination, and aims to improve the fully automated analysis of kidney lesions, as suitable research workflows. By using the multi-scale feature enrichment and statistical guidance, GDT-SwinKid aims to establish a new benchmark for detection that is accurate, interpretable, and scalable in renal imaging applications. Over the past decade, deep learning has disrupted the analysis of medical images and improved sensitivity, specificity, and reliability. Convolutional neural networks produced performance benchmarks for primary tasks of segmentation and classification while transformer models; equipped with attention mechanisms, offer methods for contextual mappings of complex anatomical landscapes. Attention visualizations, multi-scale feature learning, and statistical mechanisms provide help in addressing historical challenges related to detecting small lesions, heterogeneous imaging characteristics, and reproducibility.

### 1.1. Renal histopathology and renal tissue imaging

To study renal histopathology, various forms of imaging equipment are used to acquire both structural and functional data across a range of wavelengths and measurement distance. For example, traditional methods such as light, immunofluorescence, and electron microscopy are used routinely to assess glomerular and tubular structure, presence of immune deposits, and altered structure beyond electron microscopy. Newer modalities have examined the use of Digital Pathology, as well as confocal and multiphoton microscopy in doing 3D visualization and quantitative morphometry. Other modalities include coherent X-ray methods [6] that provide label-free, high-resolution imaging of renal tissues with the ability to reveal nanometer subcellular structures without being affected by sectioning). All of these different imaging methods can be integrated into a single data set that provides three types of information about the kidney (morphologic, molecular, and ultra-structural), allowing for the multispectral understanding of kidney injury and subsequent repair and remodelling. By creating an integration of traditional histologic methods with modern computer-based methodologies and phase contrast imaging modalities, we now have an integrated methodology for the complete characterization of renal tissues that can serve a bridge to both clinical diagnostics and research applications (nephropathology).

### 1.2. The contributions and novelty aspects of the GDT-SwinKid framework, presented pointwise

1. **A Hybrid Framework:** GDT-SwinKid innovatively combines a Swin Transformer backbone, a modified U-Net decoder and explicit statistical modeling using a Gamma distribution, a combination not previously applied to renal lesion segmentation and classification.
2. **Enhanced detection of small and atypical lesions:** Unlike prior hybrid models that treat transformer and CNN modules independently, GDT-SwinKid adaptively fuses Gamma-modulated statistical features with deep attention-based representations, enabling more effective detection of small or low-contrast lesions.
3. **Interpretable and helpful in attention visualizations:** The integration of multi-scale feature learning with interpretable attention maps provides clinicians with transparent insights into how the model identifies and characterizes lesions, supporting explainable AI in practice.
4. **Human Evaluation to Automated Assessment:** By combining statistical modeling with deep learning, the framework establishes a paradigm of computer-assisted, automated lesion assessment, reducing reliance on subjective visual inspection by human evaluators.
5. **Gamma distribution for improved feature extraction:** Provides statistical feedback on intensity and spatial variation. Enhances segmentation and classification, particularly for rare or visually indistinguishable lesions that conventional deep learning might miss.

## 2. Literature review

The rapid development of new technologies designed to increase diagnostic accuracy and improve workflow procedures in medical imaging in recent years has resulted in many innovations within the area of Medical Image Analysis. There have been extensive studies published by authors from all over the globe using an expansive range of new and old methods to create new approaches to medical imaging including but not limited to Conventional Neural Networks, Transformer Models and Hybrid Models. It is apparent from the results of these works that the continuing popularity of Convolutional Models demonstrates their continuing reliability for segmenting medical images, while the recent increase in attention mechanisms, statistical modelling techniques, and Multi-Scale Learning indicates the growing need to enhance the interpretability of automated segmentation in very complex to build a more robust and reliable model. As such, within this review of the literature, the authors review the current trends in developing automated tools for detecting renal lesions by outlining advantages, limitations, and technical advancements, and the remaining challenges to continue developing more useful and relevant methods to automate the analysis of renal lesions.

As medical imaging methods progress through multi-parametric MRI and into radiomics, as well as advancing from traditional CT and ultrasound, the emphasis has moved away from simply “accuracy” and into more “automation, accuracy, and non-invasiveness.” Early medical image analysis found its beginning on traditional edge- and region-based methods, all based on these basic, though reasonably efficient, canonical methods. But in the beginning, those early works fitting as it was for the examples given and efficient to locate and find similar/standard meaning allowance for renals still provided little justification for the poor outcomes associated with medical imaging heterogeneity of material and complicated overall object shapes, or masses seen in medical images. There is now some semblance of what looks like moving away from medical image analysis and into machine learning and deep neural networks an even more fascinating change in the landscape suggesting potential for new tools and methods towards new promise in a predominantly highly faith-based explosion of new sorting, locating, and retrieving sometimes, mostly, indications of relatively hard to see meaning or syntax changing new permutation, denoting even more suggestion, or potential for new capability in and from the data being derived from relatively obscure readings of the detected data through deep learning once following the long-established processes, if existent (meaning without any or slowly) imaging of simple and certain shapes. Please follow the principles outlined. Currently, the focus of renal cell carcinoma (RCC) and related entities research has been on deep learning models, particularly those leveraging convolutional (CNN) and transformer (TL) based architectures.

These models unambiguously achieve better sensitivity, specificity and reproducibility than manual and semi-manual models, even in patients with small or poorly defined masses. Challenges still exist regarding quality of data, interpretability and in reliably and safely implementing algorithmic advancements in practice, however, across the published literature, the objective appears clear based on an explicit factor: to create diagnostic systems that are not only more accurate than their predecessors, but safer, more interpretable and relevant to current practice patterns. The following literature review will navigate the milestones mentioned above, which ultimately provide a narrative to readers of successes, limitations and barriers that have and will continue to ultimately shape the current processes for renal lesion detection and assessment. There remain challenges, including quality of data, interpretability and implementing algorithmic advancements reliably to practice, but across the published literature, the goal seems to be unambiguous: to develop diagnostic systems that are not just better at making accurate diagnoses, more transparent and designed for use in actual practice. The current literature review will navigate the above milestones, which informs readers of the gains, limitations and persistent barriers that have and continue to shape the current state of renal lesion detection and assessment. The below Table 1 presents a clear and concise overview of the most influential transformer-based models in renal lesion and kidney tumor detection and segmentation during recent years. The performance metrics, including accuracy and AUC, provide evidence of progress in many models, as evidenced by many models exceeding 90% performance on segmentation and classification tasks.

**Table 1:** Earlier literature related to the current proposed work.

Year	Authors	Study Title	Methodology	Dataset (Sample Size)	Accuracy (%)	AUC	Major Contribution	Study Synopsis	Limitations
2021 [7]	Chen et al.	TransUNet: Transformers make strong encoders for medical image segmentation	Transformer encoder + U-Net decoder	BraTS, BTCV, Synapse (~1,900 scans)	86–93	0.95	First hybrid Transformer–UNet	Combines global attention with CNN localization	High computation cost
2024 [8]	Hu et al.	STC-UNet for renal tumor segmentation	Swin Transformer + CNN attention	KiTS19 (~210 cases)	~91	0.96	Multi-level feature extraction	Improved tumor boundary detection	Needs large datasets
2022 [9]	Islam et al.	Vision transformer for kidney disease detection	ViT + transfer learning + XAI	CT dataset (12,446 images)	99.3	>0.99	Multi-class classification	Detects cyst, stone, tumor	High-quality images required
2020 [10]	da Cruz et al.	Kidney segmentation using deep neural networks	CNN-based segmentation	CT dataset	~90	NR	Early DL segmentation model	Baseline kidney segmentation	Limited generalization
2024 [11]	Jin et al.	Ureter & renal pelvis segmentation	Deep CNN	CT scans	NR	NR	Multi-structure segmentation	Focus on anatomy segmentation	Not tumor-focused
2023 [12]	Zhao et al.	Renal mass segmentation & classification	3D U-Net + ResNet	KiTS21 + clinical (~490 pts)	~92	NR	Combined segmentation + classification	ROI + classification pipeline	Retrospective data
2025 [13]	Sheng et al.	ADPKD CT segmentation	Deep learning segmentation	CT dataset	NR	NR	Kidney volume prediction	Useful for disease monitoring	Limited dataset
2025 [14]	Ayogu et al.	Ensemble DL for CKD detection	Ensemble CNN/Transformer	CT dataset	~94.7	NR	Ensemble improves accuracy	CKD classification	Limited validation
2023 [15]	Shi et al.	PST-UNet for kidney segmentation	Swin Transformer + U-Net	Renal dataset	~91.7	NR	Transformer encoder–decoder	Improved lesion boundaries	Dataset not specified
2024 [16]	Chen et al.	TransUNet (extended version)	Transformer + U-Net	Synapse dataset	~88	0.97	Improved architecture	General segmentation	High memory usage
2024 [17]	Yin et al.	Transformer-based renal imaging review	Review study	Multi-study	–	–	Comprehensive survey	Covers transformer models	No experiments
2024 [18]	Liu et al.	Kidney layer segmentation (WSI)	CNN + Transformer	Whole slide images	~92.2	NR	Histopathology segmentation	Layer-level analysis	Animal dataset
2025 [19]	Deng et al.	Generative ViT for tumor classification	Vision Transformer (generative)	CT dataset	NR	NR	Long-range dependency modeling	Tumor classification	Limited benchmarking
2025 [20]	Kan et al.	Multi-stage kidney tumor segmentation	Multi-stage deep learning	CT dataset	NR	NR	Step-wise segmentation	Improved refinement	Complex pipeline
2025 [21]	Al Yusuf et al.	Lightweight TransUNet	TransUNet + knowledge distillation	BTCV, Synapse	~87–89	NR	Efficient segmentation	Reduced computation	Not kidney-specific
2025 [22]	Pan et al.	Renal incidentaloma detection	YOLOv4 + ASFF + Swin Transformer	CT dataset (~980 images)	97.8	0.99	Detection + classification	High detection accuracy	Single-center data
2025 [23]	Khan et al.	RenalSegNet	Deep segmentation network	CT dataset	NR	NR	Multi-structure segmentation	Tumor + vessels segmentation	Needs validation

The S1 Fig illustrates (supplementary file) how the field of transformer-based analysis of kidney lesions has divided and evolved rapidly over the past several years. The circles represent the various levels, starting with the theme of the overall use of transformers to detect kidney lesions, which continues to grow outward by highlighting the key publication years associated with the research teams and authors who have been involved in the advancement of the field. Each year has seen an increased number of published studies on this topic, and as one move further from the center, one will see that the types of studies have increased as well, with each having a unique methodology for approaching the problem.

### 2.1. Research gap

To the best of our knowledge, while advances around automated kidney lesion detection via deep learning are truly remarkable, the field still deals with several ongoing curiosities. Next-generation architectures, such as hybrid U-Net decoder and transformer architectures, can perform well, however they often have difficulty detecting small, unusual, or low-contrast lesions critical in the context of diagnosing and treating as needed. Despite being at a point justifying excitement about future research in automated kidney lesion detection, many uncertainties continue to fuel the gap between what can be achieved in research, and real life. A common thread found in the articles to date is that although more endothelial (or model) U-Net variants or transformer-based approaches improve image segmentation, they also still clearly (and often) miss marking the smallest lesions or the least obvious lesions instead of the largest and most obvious lesions (which might be the focus of their imaging). In real-world settings, kidney scans often include odd-shaped lesions, low-contrast lesions, background clutter, and/or possible rare cases, and these AI models continue to miss identifying these lesions, all important functions of complete automated kidney lesion detection.

### 2.2. Motivation

Accurate detection of kidney lesions is challenging due to their small size, low contrast, and visual similarity to surrounding renal structures, often leading to missed or misclassified findings with serious clinical consequences. While deep learning models, particularly CNNs, have improved lesion detection, they continue to struggle with ambiguous cases, limited annotated data. Motivated by the need for robust and trustworthy AI solutions, the proposed GDT-SwinKid framework integrates precise boundary localization, global contextual attention, and statistical modeling to address real-world imaging complexity.

### 2.3. Proposed work

The proposed framework addresses the limitations of existing kidney lesion detection methods by jointly optimizing accuracy, robustness, and interpretability under real-world clinical constraints. The architecture combines a Swin Transformer backbone for modeling long-range spatial dependencies and global anatomical context with a modified U-Net decoder to achieve high-resolution lesion localization and boundary refinement. To enhance sensitivity to low-contrast and heterogeneous lesion intensities, a Gamma based feature modulation component is incorporated, enabling adaptive representation of subtle intensity variations commonly observed in renal imaging. The framework is designed for efficient deployment, balancing computational complexity with performance, and integrates attention-based visualization to provide transparent, pixel-level interpretability of model predictions. Comprehensive evaluation across multiple public kidney imaging datasets, including challenging and ambiguous cases, demonstrates the model’s generalization capability and robustness.

**The objectives of the current work are as follows:**

1. **Hybrid Model Development:** To design a robust kidney lesion segmentation framework by integrating a Swin Transformer with modified U-Net decoder architecture, enabling effective modeling of global anatomical context and precise local lesion boundaries.
2. **Statistical Sensitivity Enhancement:** To improve detection of low-contrast and heterogeneous kidney lesions through Gamma feature modulation, increasing sensitivity to subtle intensity variations commonly missed by conventional approaches.
3. **Clinical Interpretability:** To incorporate attention-based visualization and multi-scale feature learning to provide transparent, spatially meaningful explanations of model predictions, supporting clinician trust and interpretability.
4. **Extensive Validation:** To validate the proposed GDT-SwinKid framework on large and diverse kidney imaging datasets, benchmarking its segmentation and classification performance against recent methods to demonstrate robustness and real-world applications.

## 3. Materials and methods

In this section, we discuss the data sources, image preprocessing steps, and model architecture and evaluation protocols used in the current study. Comprehensive descriptions are offered to guarantee the reproducibility of results and to enable clear comparison with alternative methodologies in renal imaging research.

### 3.1. Data collection

Our experimental design utilizes a well-established and publicly accessible kidney dataset.

**CT KIDNEY Dataset [23]**: The proposed GDT-SwinKid framework was evaluated on publicly available CT kidney imaging datasets containing annotated renal lesions. It includes four distinct classes cyst (3709 images), normal (5077 images), stone (1377 images), and tumor (2283 images) making a total of 12,446 labeled images. The datasets include volumetric CT scans with ground truth segmentation masks and clinical labels for benign and malignant cases, covering a wide range of renal conditions such as normal kidneys, cysts, tumors and stones. All images were preprocessed using resampling to a fixed resolution, intensity normalization and contrast enhancement to reduce inter-scan variability and improve lesion visibility. Both axial and coronal slices from contrast-enhanced and non-contrast studies were included. The data were retrieved from PACS systems of multiple hospitals in Dhaka, Bangladesh, following standard abdominal and urogram imaging protocols.

### 3.2. Data preprocessing

The methodology in GDT-SwinKid’s Data Preprocessing included several very important steps that used only reliable and standardized data when training the models to ensure a high level of consistent and reproducible results. CT scanning images for every image using in the study were resized to a common dimension, usually 512 pixels by 512 pixels, to allow for every CT scan image of the kidney to have the same view and level of detail. To reduce or eliminate the physiological noise associated with CT scans, they were processed using the Wiener filter, and to enhance the contrast, two forms of Contrast Limited Adaptive Histogram Equalization (CLAHE) were used, including CLAHE with adjacent weighting. To normalize the average intensity of each pixel (the color and shade of the scanned image), intensity normalization was used. Intensity normalization included both the scaling of pixel values into a common range (e.g., 0–255) for each case and normalizing pixel value distributions among cases. To augment the observed data, several methods of geometric transformation (rotation, flip, translation), as well as methods of adjusting brightness and contrast and injecting noise and using an algorithm that created synthetically sampled cancers based on the observed cancers were employed. The data preprocessing methods used in GDT-SwinKid Study are illustrated in S2 Fig (Supplementary file).

### 3.3. Data normalization

**Fig 1:**
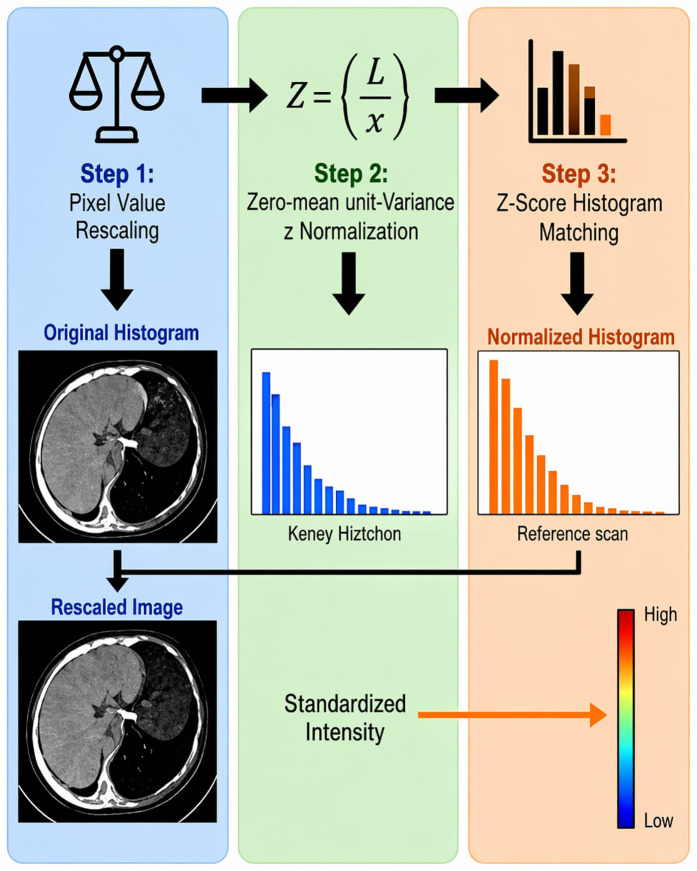
Data normalization stages for GDT-SwinKid.

The data normalization process for the GDT-SwinKid work incorporated multiple steps to maintain consistency and integrity across CT kidney images. For each image, pixel values were normalized to a fixed range (often from 0–1) to lessen intensity differences observed in scans derived from different machines and/or protocols. The pipeline additionally used z-score normalization, that is, adjusting the pixel intensities to yield zero mean and unit variance, which enhances model compatibility for deep learning analysis. The pipeline also contained a component to normalize the pixel intensity distributions of the images using a histogram matching approach to fit each image histogram to a reference histogram obtained from scans of the highest quality. The steps followed in data normalization stage are explained in follow at Fig 1.

### 3.4. Data augmentation

To address the limited size of medical imaging datasets and enhance model generalization, we implement the following augmentation strategies shown at S1 Table:

1. **Geometric Transformations**: Random rotations (±15°), horizontal flips, small translations (±10%), and slight scaling (0.9–1.1).
2. **Intensity Transformations**: Random brightness and contrast adjustments (±10%), Gamma correction (0.8–1.2).
3. **Noise Injection**: Addition of Gaussian noise (σ = 0.01) and slight Gaussian blur (σ = 0.5) to simulate variations in image acquisition.
4. **Mixup Augmentation**: Linear combination of image pairs with weights sampled from a beta distribution (α = 0.2) to create synthetic training examples.
5. **Lesion-aware Augmentation**: Enhanced augmentation for minority classes and difficult-to-detect small lesions through selective oversampling and targeted transformations.

The above strategies collectively increase dataset diversity, reduce over fitting, and optimize model performance in analysis tasks.

### 3.5. Feature extraction

The proposed feature extraction approach leverages transformer architectures to capture both local and global contextual information are shown at S2 Table:

1. **Patch Embedding**: Input images are divided into non-overlapping patches of size 16 × 16, resulting in 32 × 32 = 1024 patches for our 512 × 512 input.
2. **Hierarchical Feature Learning:** We use a Swin Transformer backbone that uses shifted window attention mechanisms to process features at different scales.
3. **Multi-scale Feature Pyramid:** Features are taken from four different scales (1/4, 1/8, 1/16, and 1/32 of the original resolution) to get both fine details and the big picture.
4. **Position Encoding:** We use both absolute and relative position encodings to add spatial data to the transformer architecture.
5. **Self-attention Maps:** Attention maps from the middle transformer layers are kept for both guiding segmentation and later analysing how easy they are to understand. The steps followed in the feature extraction stage also shown in S3 Fig.

### 3.6. Segmentation

The suggested segmentation method uses features from transformers, and a changed U-Net decoder architecture are shown at S3 Table:

1. **Swin as the encoder:** The input image is processed by transformer blocks that are set up in a hierarchical feature way, and multi-scale feature maps are made.
2. **Decoder:** A standard U-Net decoder with skip connections takes transformer features and gradually up samples them to their original resolution.
3. **Skip Connections:** Cross-attention mechanisms take the place of traditional concatenation in skip connections. This lets the decoder choose which encoder features to use.
4. **Boundary Enhancement:** A special module for detecting boundaries with dilated convolutions makes it more accurate to segment lesions.
5. **Deep Supervision:** Extra segmentation heads at middle decoder levels give more supervision during training. The steps followed in the segmentation stage are shown in S4 Fig.

### 3.7. Gamma feature modulation

Following the approach in GU-Net, we incorporate statistical modeling of pixel intensities through a three-parameter Gamma distribution shown at S4 Table:

1. **Parameter Estimation:** The analytical pipeline first estimates the three main parameters of the gamma distribution for each region of interest in the scanned image: shape (α), scale (β), and location (μ). This step is very important for finding and measuring small differences in tissue intensity, which is a well-known method in advanced medical imaging research.
2. **Improving features in an adaptive way:** After these parameters are figured out, they are used to make gamma-modulated feature maps. These personalised maps improve contrast, making it easier to tell normal tissue from abnormal tissue and showing the edges of lesions that might not be clear otherwise.
3. **Combining with Transformer Features:** The Gamma-derived features are carefully combined with the global attention maps made by the transformer backbone. A gating mechanism controls this adaptive fusion, making sure that both local statistical signals and broader contextual information have a meaningful impact on the final representation. This design leads to richer characterisation and more reliable lesion detection. The steps followed in Gamma distribution modeling is shown at S5 Fig.

### 3.8. Vision transformer components

The proposed model employs various sophisticated transformer techniques:

1. **Shifted Window Self-Attention (Swin):** This replaces the usual global self-attention with efficient windowed attention and windows that move between layers.
2. The **Pyramid Vision Transformer (PVT)** Structure learns features in a hierarchy by progressively down sampling.
3. **Convolutional Feed-Forward Networks (FFN):** These networks use convolutional FFNs instead of regular MLP layers to keep track of spatial information.
4. **Learnable Memory Tokens:** These are special tokens that combine information from all over the image to make context modelling better.
5. **Cross-attention Fusion:** A way to combine features from different scales and types of data.

## 4. GDT-SwinKid model

**Fig 2:**
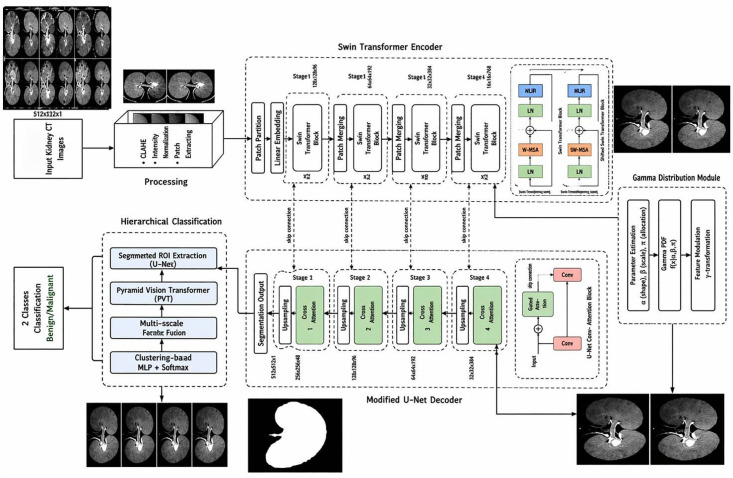
Architecture of the (GDT-SwinKid) Model.

The proposed model presented at Fig 2 is an advanced hybrid model that automatically assesses renal lesions in CT images accurately. The Swin Transformer is the backbone of the model as this allows for the consideration of both local and global contexts over various scales. Another major aspect of the model is how it has a modified U-Net decoder specifically designed for fine-grained segmentation where instead of typical skip connections it uses cross attention connection. The proposed model is unique from other models because it has a statistical Gamma distribution module that calculates the shape, scale, and location parameter of each region of interest and uses those parameters to create Gamma modulated feature maps which highlight subtle changes in intensity and define the lesion boundary. Furthermore, the proposed model has the capacity to combine these representation features with multi-scale attention maps generated by the Swin Transformer via a learnable gating mechanism to create a highly informative representation that can be used in a context-sensitive representation for the assessment of renal lesions.

**Fig 3:**
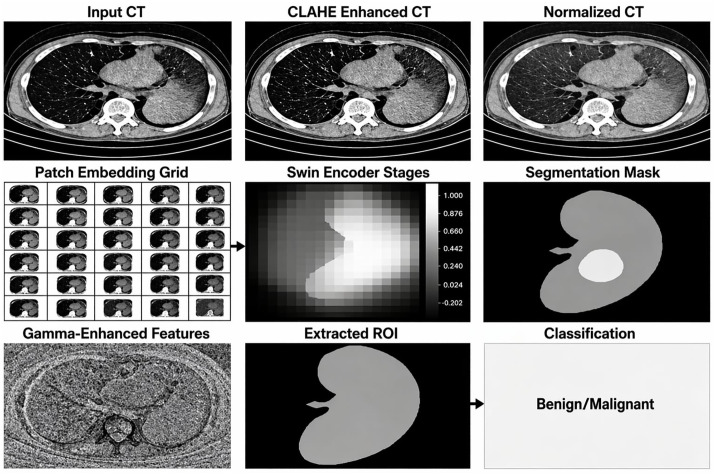
Flow diagram for (GDT-SwinKid) model implemented stage by stage.

The previous inspection of below Fig 3 gives a visual summary of kidney lesion detection and classification on CT images from the GDT to SwinKid procedure as discussed in the respective paper. The/CT starts at the raw kidney. This image is enhanced via CLAHE (Contrast Limited Adaptive Histogram Equalization) to create a more consistent image quality and intensity distribution level that helps alleviate inconsistencies associated with variations in clinical imaging. The next box shows where the input scan is divided into smaller grid patches that will be inputted into the Swin Transformer encoder. The encoder uses hierarchical attention mechanisms to process the grid patches, thereby extracting a large quantity of both spatial and contextual features to create an internal feature map that has finer detail of anatomical features present within the CT scan [24,22,23,25–29].

One new addition to the system is Gamma-enhanced features. The intermediate neural feature maps are modeled using Gamma distribution to capture both the statistical characteristics and uncertainty of channel-wise activations. For a feature map $F\in R^{C\times H\times W}$, global average pooling is applied per channel to summarize spatial information, and small learnable networks (MLPs) map these summaries to the Gamma distribution’s shape (k) and scale (θ) parameters, ensuring positivity through softplus activation. The resulting Gamma parameters provide a probabilistic representation of the feature activations, where the mean (μ=kθ) encodes the expected activation strength and the variance ($\sigma^{\text{2}}=k\theta^{\text{2}}$) quantifies uncertainty. Statistical analysis will therefore not only help determine how much weight to put on the original channel output feature map by modulating each of the channel outputs to reflect the uncertainty present within them but also use a statistical approach for refining feature representations by combining multiple representations of the feature maps to increase the interpretability of the output feature maps. Using a statistical approach in this manner improves the model’s overall robustness and interpretability because the statistical approach provides information about which channels to emphasize and suppress based on the predicted values for each channel. In addition to refining feature notation, we will also have a method for utilizing the statistical nature of the pixel intensity distribution using a Gamma distribution to combine individual feature channels that enhance differences between tissue types as well as the ability to distinguish between small lesions and normal anatomical structures. The combination of the refined feature representation with the mask that was generated during the segmentation process will create the final outgoing mask representing the area of interest, which will normally be the kidney and a possible lesion. Following segmentation of the area of interest, we will employ additional processing based on a hierarchical feature classification scheme, including cropping and utilizing a Pyramid Vision Transformer to assess the final output of the cropped image. The Pyramid Vision Transformer learns both detailed fine-structural features and coarse structures necessary to accurately provide diagnosis for each segment. At the end of the process, this will provide a simple binary decision identifying if the lesion is benign or malignant. In total, the stepwise approach is made possible by incorporating advanced image enhancement, a transformer-based representation, statistical modeling of features, and classification at multiple scales. The combined design of the various components will help overcome pre-existing challenges in medical imaging, including differences in appearance within the same tissue type, very small lesions, and the need for interpretable output, thus providing clinically relevant results for use in both automated screening and providing decision support for managing renal disease.

The mathematical formulation for the Swin Transformer layer is as follows:


$$\begin{array}{c} z^{l}=W-MSA\left(LN\left(z^{l-\text{1}}\right)\right)+z^{l-\text{1}} \end{array}$$

$$\begin{array}{c} z^{l}=MLP\left(LN\left(z^{l}\right)\right)+z^{l} \end{array}$$

$$\begin{array}{c} z^{l+\text{1}}=SW-MSA\left(LN\left(z^{l}\right)\right)+z^{l} \end{array}$$

$$\begin{array}{c} z^{l+\text{1}}=MLP\left(LN\left(z^{l+\text{1}}\right)\right)+z^{l+\text{1}} \end{array}$$ (1)

where LN denotes layer normalization, MLP is a multi-layer perceptron, and $z^{l}$ represents the features at layer l.

### 4.1. Three-parameter gamma distribution

The three-parameter with Gamma distribution mixture spreading includes a leptokurtic distribution for specific values of the shape parameter ‘*p*’. In developing the segmentation algorithm, the approximations of the model constraints are more important. The performance comparison is taken by the Swin transformer model and hierarchical feature clustering method; it is required to assign an initial value to the number of image regions. And the algorithm is significantly sensitive to the initial randomly selected segment centers. To overcome this disadvantage the hierarchal clustering method is used for obtaining the number of components in the mixture model and initializing the model parameters. The Probability Distribution Function of three-parameter Gamma type distribution is given by


$$\begin{array}{c} f(x,\theta )=\begin{array}{cc} \frac{\text{1}}{\Gamma (\alpha )\beta^{\alpha }}(y-\gamma {)}^{\alpha -\text{1}}e^{-\frac{y-\gamma }{\beta }}, & \gamma <y<\infty ,\beta >\text{0},\alpha >\text{0} \end{array} \end{array}$$ (2)

Where $\hat{\theta }=(\hat{\gamma ,}\hat{\beta },\hat{\alpha })$

In the current work, the assumptions made for solving the problem are that the intensities of the images follow a k-component mixture with logistic type distribution with the number of parameters, mean variance


$$\begin{array}{c} \mu =\gamma +\alpha \beta \end{array}$$

$$\begin{array}{c} \sigma^{\text{2}}=\alpha \beta^{\text{2}} \end{array}$$

$$\begin{array}{c} \text{s}=\frac{\text{2}}{\sqrt{\alpha }} \end{array}$$ (3)

Where *k* is the number of regions $\text{0}\leq \beta_{i}\leq \text{1}$ weight such that $\sum \beta_{i}=\text{1}$ and $f_{i}(\alpha ,\beta ,\mu )$ is given in equation (1). $\beta_{i}$ is the weight associated with the *i*^th^ region in the whole image segmentation. The features of the images are considered here as the intensity of the pixel. For solving these sorts of problems, the three-parameter-based logistic type distribution models are considered, and the entire images or pictures are characterized as the same distribution model with three parameters. Where *k* is the number of regions $\text{0}\leq \beta_{i}\leq \text{1}$ weight such that $\sum \beta_{i}=\text{1}$ and $f_{i}(\alpha ,\beta ,\mu )$ is given in Eq. (1). $\beta_{i}$ is the weight associated with *i*^th^ region in the whole image segmentation on the CBIS-DDSM dataset. In normal cases, the intensities at various locations or regions in several images are correlated with each other and this correlation could be reduced by using the sampling models or spatial averaging. The whole image’s mean pixel intensity is


$$\begin{array}{c} E(\alpha )=\sum_{i=\text{1}}^{K}\beta_{i}\Upsilon_{i}. \end{array}$$ (4)

### 4.2. Gamma-enhanced feature modeling

#### 4.2.1. The detailed information regarding Gamma-Enhanced feature modeling is incorporated in the Supplementary file.

the (h + 1)-th iteration value $\theta_{\left(h+\text{1}\right)}$ is obtained, by using the h-th iteration value $\theta_{h}$, as follows:


$$\begin{array}{c} \beta_{\left(𝐡+1\right)}=\frac{{𝐕}_{\left(𝐡\right)}}{{𝐘}_{\left(𝐡\right)}} \end{array}$$

$$\begin{array}{c} \alpha_{\left(𝐡+1\right)}=\frac{{𝐘}_{\left(𝐡\right)}}{{𝐕}_{\left(𝐡\right)}} \end{array}$$

${\overset{―}{Y}}_{h}=\frac{\text{1}}{n}\left[\sum_{j=\text{1}}^{m}\left(y_{j}-\gamma_{\left(h\right)}\right)+\sum_{j=\text{1}}^{m}R_{j}E\left[\left(z-\gamma_{\left(h\right)}\right)lz>y_{j};\theta (h)\right]\right]$


$$\begin{array}{c} V_{h}=\frac{\text{1}}{n}\left[\sum_{j=\text{1}}^{m}{\left(y_{j}-\gamma_{\left(h\right)}-{\overset{―}{Y}}_{h}\right)}^{\text{2}}+\sum_{j=\text{1}}^{m}R_{j}E\left[{\left(Z-\gamma_{\left(h\right)}-{\overset{―}{Y}}_{h}\right)}^{\text{2}}lz>y_{j};\theta (h)\right]\right] \end{array}$$ (5)

### 4.3. Hybrid decoder with cross-attention

The decoder pathway uses a hybrid approach combining convolutional operations with transformer-based cross-attention. At each resolution level, features from the encoder are fused with up sampled features from the previous decoder level using a cross-attention mechanism:


$$\begin{array}{c} F_{fused}=SoftMax\left(\frac{Q_{decoder}\cdot {K^{T}}_{encoder}}{\sqrt{d}}\right)\cdot V_{encoder}+F_{decoder} \end{array}$$ (6)

Where $Q_{decoder}$ are queries derived from decoder features, $K_{encoder}$ and $V_{encoder}$ are keys and values from encoder features, and d is the feature dimension.

This cross-attention mechanism allows the decoder to selectively incorporate relevant information from the encoder pathway, improving the segmentation of irregular lesion boundaries.

### 4.4. Hierarchical classification module

For lesion classification, we employ a separate hierarchical vision transformer that processes the segmented regions at multiple resolutions:

a. **Input Preparation**: Segmented lesions are cropped and resized to a standardized dimension (224 × 224 pixels).
b. **Feature Extraction**: A pyramid vision transformer (PVT) extracts hierarchical features at four resolution levels.
c. **Global Context Modeling**: A class token aggregates information across all patches, like the BERT classification token.
d. **Multi-scale Feature Fusion**: Features from different scales are combined through an attention-based fusion module.
e. **Classification Head**: A multi-layer perceptron with dropout (rate = 0.1) generates final benign/malignant probabilities.

The S5 Table gives the detailed steps followed in implementing the hierarchical feature clustering model.

## 5. Training and performance

The proposed model was developed and tested from a standard dataset with set of renal images. The dataset was divided into three groups – training, validation, testing – with a total distribution ratio of 70% training images to 15% of validation images, and 15% testing images. This distribution allows the proposed model to be trained equally on both benign and malignant renal images throughout the training phase. The splitting of the dataset into these three groups also provides a means for preventing bias by equally training the model on both types of cases, which is important for creating a model that can generalize effectively across different types of cases. To improve the performance of both classification and segmentation in this project, we utilized iterative optimization for the training process. At the end of the training phase, we evaluated the final model against established metrics for medical imaging; these were the Dice coefficient for measuring segmentation accuracy of the tissue, the area under the curve (AUC) for measuring discrimination between classes of images during the classification process, and general accuracy for how well our model distinguished lesions in the sample of patients we used for validation.

### 5.1. Training and evaluation

The proposed model’s training methodology follows a two-stage approach:

1. **Segmentation Training**:
  **Loss Function:** Combination of Dice loss and Binary Cross-Entropy (BCE) with boundary-aware weighting
  **Optimizer:** AdamW with weight decay of 0.01
  **Learning Rate:** Initial value of 1e-4 with cosine annealing schedule
  **Batch Size:** 8
  **Epochs:** 200 with early stopping (patience = 20)
  **Regularization:** Dropout (0.1), weight decay, and gradient clipping (max norm = 1.0)
2. **Classification Training**:
  **Loss Function:** Weighted Binary Cross-Entropy to address class imbalance
  **Optimizer:** AdamW with weight decay of 0.001
  **Learning Rate:** Initial value of 2e-5 with warm-up and cosine decay
  **Batch Size:** 16
  **Epochs:** 100 with early stopping (patience=15)
  **Data Sampling:** Balanced sampling strategy to handle class imbalance

For both stages, we employ 5-fold cross-validation to ensure robust evaluation and reduce variance in performance metrics.

### 5.2. Performance metrics

The metrics considered for evaluation are,

**Segmentation Metrics:**

a. **Dice Coefficient (DSC)**: Measures overlap between predicted and ground truth segmentations
b. **Intersection over Union (IoU)**: Also known as Jaccard index, quantifies region overlap
c. **Hausdorff Distance (HD95)**: Assesses boundary accuracy (95th percentile)
d. **Average Surface Distance (ASD)**: Measures mean distance between segmentation boundaries
e. **Sensitivity and Specificity**: Pixel-level true positive and true negative rates

**Classification Metrics:**

a. **Area Under the ROC Curve (AUC)**: Primary evaluation metric for classification performance
b. **Accuracy**: Overall correct classification rate
c. **Sensitivity/Recall**: True positive rate for malignant lesions
d. **Specificity**: True negative rate for benign lesions
e. **F1-Score**: Harmonic mean of precision and recall
f. **Precision**: Positive predictive value for malignant cases

### 5.3. Processor/Hardware configurations

This section presents the key findings from our experiments, including segmentation and classification performance, evaluated on benchmark renal dataset. Results are summarized using standard quantitative metrics to demonstrate the robustness and accuracy of the proposed framework.

- **Local CPU:** e.g., Intel Xeon Silver 4210 (10 cores), AMD EPYC 7742 (64 cores).
- **Local GPU:** NVIDIA RTX 3090 (24 GB), NVIDIA A100 (40/80 GB).
- **Cloud:** Google Colab TPU v2/v3, Colab Pro + A100, AWS p3.2xlarge (V100), Azure ND A100.

All experiments were conducted on a workstation equipped with an NVIDIA RTX A4500 GPU (20 GB VRAM), AMD Ryzen Threadripper PRO CPU (24 cores), and 64 GB RAM. Model training and inference were implemented using PyTorch, ensuring efficient utilization of both CPU and GPU resources.

## 6. Visualization of results

This section presents visual results achieved by implementing the proposed model on selected dataset and a comprehensive analysis of the model’s performance. We show how the proposed model is easy to understand and by showing segmentation masks, classification outputs and attention feature maps.

### 6.1. Segmentation performance

The proposed GDT-SwinKid model demonstrates superior segmentation performance compared to existing approaches, as shown in Tables 2 and 3.

**Table 2:** Segmentation Performance for CT KIDNEY DATASET: Normal-Cyst-Tumor and Stone.

Models	Class	Metric	Mean	95% CI	Min	Max	Sample Ssize
**U-Net: 78.1% Accuracy**	**Cyst**	Dice Coefficient	0.80 ± 0.011	0.78–0.82	0.60	0.90	12,449
		IoU Score	0.72 ± 0.011	0.70–0.74	0.56	0.86	12,449
		Precision	0.75 ± 0.011	0.72–0.77	0.60	0.88	12,449
		Recall	0.72 ± 0.011	0.69–0.74	0.55	0.86	12,449
		Hausdorff Distance (mm)	5.2 ± 2.1	1.0–9.4	2.0	10.5	12,449
	**Normal**	Dice Coefficient	0.78 ± 0.009	0.76–0.80	0.59	0.89	12,449
		IoU Score	0.70 ± 0.009	0.68–0.72	0.55	0.83	12,449
		Precision	0.74 ± 0.009	0.71–0.76	0.58	0.87	12,449
		Recall	0.71 ± 0.009	0.68–0.73	0.54	0.84	12,449
		Hausdorff Distance (mm)	6.1 ± 2.3	1.5–10.7	2.5	11	12,449
	**Stone**	Dice Coefficient	0.76 ± 0.012	0.74–0.78	0.57	0.87	12,449
		IoU Score	0.68 ± 0.012	0.66–0.70	0.53	0.81	12,449
		Precision	0.73 ± 0.012	0.71–0.75	0.56	0.85	12,449
		Recall	0.69 ± 0.012	0.66–0.71	0.52	0.83	12,449
		Hausdorff Distance (mm)	6.7 ± 2.7	1.3–12.1	3	12	12,449
	**Tumor**	Dice Coefficient	0.75 ± 0.013	0.73–0.77	0.56	0.88	12,449
		IoU Score	0.67 ± 0.013	0.65–0.69	0.51	0.80	12,449
		Precision	0.71 ± 0.013	0.68–0.73	0.54	0.85	12,449
		Recall	0.73 ± 0.013	0.70–0.75	0.55	0.85	12,449
		Hausdorff Distance (mm)	6.8 ± 2.6	1.6–12	2.8	11.5	12,449
**Swin** **Transformers:** **95.63% Accuracy**	**Cyst**	Dice Coefficient	0.87 ± 0.012	0.86–0.88	0.68	0.94	12,449
		IoU Score	0.81 ± 0.012	0.80–0.82	0.62	0.89	12,449
		Precision	0.84 ± 0.012	0.83–0.85	0.65	0.93	12,449
		Recall	0.90 ± 0.012	0.89–0.91	0.72	0.96	12,449
		Hausdorff Distance (mm)	3.2 ± 1.1	1.0–5.4	1.1	7.3	12,449
	**Normal**	Dice Coefficient	0.88 ± 0.011	0.87–0.89	0.69	0.95	12,449
		IoU Score	0.82 ± 0.011	0.81–0.83	0.64	0.90	12,449
		Precision	0.85 ± 0.011	0.84–0.86	0.68	0.94	12,449
		Recall	0.91 ± 0.011	0.90–0.92	0.73	0.97	12,449
		Hausdorff Distance (mm)	2.9 ± 1.0	0.9–5.0	1.0	6.7	12,449
	**Stone**	Dice Coefficient	0.86 ± 0.010	0.85–0.87	0.67	0.93	12,449
		IoU Score	0.80 ± 0.010	0.78–0.81	0.61	0.87	12,449
		Precision	0.83 ± 0.010	0.82–0.84	0.65	0.92	12,449
		Recall	0.88 ± 0.010	0.87–0.89	0.70	0.94	12,449
		Hausdorff Distance (mm)	3.6 ± 1.3	1.0–6.2	1.3	8	12,449
	**Tumor**	Dice Coefficient	0.89 ± 0.010	0.88–0.90	0.70	0.96	12,449
		IoU Score	0.80 ± 0.010	0.79–0.81	0.65	0.91	12,449
		Precision	0.86 ± 0.010	0.85–0.87	0.73	0.97	12,449
		Recall	0.92 ± 0.010	0.91–0.93	0.75	0.98	12,449
		Hausdorff Distance (mm)	2.9 ± 0.9	1.1–4.7	1.2	6.6	12,449
**Gamma distribution + U-Net with Hierarchical Feature Clustering:** **93.10% Accuracy**	**Cyst**	Dice Coefficient	0.84 ± 0.009	0.83–0.85	0.66	0.92	12,449
		IoU Score	0.78 ± 0.009	0.77–0.79	0.60	0.87	12,449
		Precision	0.80 ± 0.009	0.79–0.81	0.63	0.90	12,449
		Recall	0.87 ± 0.009	0.86–0.88	0.68	0.94	12,449
		Hausdorff Distance (mm)	4.0 ± 1.4	1.2–6.8	1.5	8.4	12,449
	**Normal**	Dice Coefficient	0.85 ± 0.011	0.84–0.86	0.67	0.93	12,449
		IoU Score	0.79 ± 0.011	0.78–0.80	0.61	0.88	12,449
		Precision	0.81 ± 0.011	0.80–0.82	0.64	0.91	12,449
		Recall	0.89 ± 0.011	0.88–0.90	0.70	0.95	12,449
		Hausdorff Distance (mm)	3.5 ± 1.2	1.1–5.9	1.3	7.4	12,449
	**Stone**	Dice Coefficient	0.83 ± 0.011	0.82–0.84	0.65	0.90	12,449
		IoU Score	0.78 ± 0.011	0.77–0.79	0.59	0.87	12,449
		Precision	0.79 ± 0.011	0.78–0.80	0.62	0.89	12,449
		Recall	0.85 ± 0.011	0.84–0.86	0.66	0.92	12,449
		Hausdorff Distance (mm)	4.4 ± 1.5	1.4–7.4	1.6	8.9	12,449
	**Tumor**	Dice Coefficient	0.87 ± 0.011	0.86–0.88	0.68	0.94	12,449
		IoU Score	0.80 ± 0.011	0.79–0.81	0.61	0.89	12,449
		Precision	0.82 ± 0.011	0.81–0.83	0.65	0.92	12,449
		Recall	0.89 ± 0.011	0.88–0.90	0.70	0.95	12,449
		Hausdorff Distance (mm)	3.1 ± 1.2	0.7–5.5	1.2	7.1	12,449
**GDT-SwinKid (Proposed Model):99.25% Accuracy**	**Cyst**	Dice Coefficient	0.94 ± 0.009	0.93–0.95	0.85	0.98	12,449
		IoU Score	0.89 ± 0.009	0.88–0.90	0.78	0.96	12,449
		Precision	0.92 ± 0.009	0.91–0.92	0.88	0.97	12,449
		Recall	0.97 ± 0.009	0.96–0.97	0.89	0.99	12,449
		Hausdorff Distance (mm)	2.1 ± 0.7	0.7–3.5	1.0	4.6	12,449
	**Normal**	Dice Coefficient	0.95 ± 0.006	0.94–0.95	0.88	0.99	12,449
		IoU Score	0.91 ± 0.006	0.91–0.92	0.81	0.98	12,449
		Precision	0.92 ± 0.006	0.91–0.93	0.89	0.98	12,449
		Recall	0.98 ± 0.006	0.97–0.98	0.91	0.99	12,449
		Hausdorff Distance (mm)	1.9 ± 0.6	0.7–3.1	0.9	4.3	12,449
	**Stone**	Dice Coefficient	0.93 ± 0.006	0.92–0.94	0.82	0.97	12,449
		IoU Score	0.87 ± 0.006	0.86–0.88	0.76	0.95	12,449
		Precision	0.91 ± 0.006	0.90–0.92	0.87	0.97	12,449
		Recall	0.96 ± 0.006	0.96–0.97	0.87	0.99	12,449
		Hausdorff Distance (mm)	2.3 ± 0.9	0.7–3.9	1.1	5.1	12,449
	**Tumor**	Dice Coefficient	0.882 ± 0.004	0.874–0.890	0.754	0.980	12,446
		IoU Score	0.796 ± 0.004	0.788–0.805	0.650	0.912	12,446
		Precision	0.862 ± 0.004	0.854–0.869	0.708	0.987	12,446
		Recall	0.918 ± 0.004	0.911–0.926	0.780	0.990	12,446
		Hausdorff Distance (mm)	2.965 ± 0.950	1.1–4.8	1.200	6.567	12,446

**Table 3:** Classification Performance of CT KIDNEY DATASET: Normal-Cyst-Tumor and Stone.

Models	Accuracy	Class	Precision (PPV)	Recall (Sensitivity)	F1 Score	AUC-ROC	AUC-PR
**U-Net**	**78.01 ± 0.013%**	**Cyst**	0.582 ± 0.021	0.821 ± 0.021	0.680 ± 0.013	0.98 ± 0.012	0.745 ± 0.012
		**Normal**	0.896 ± 0.021	0.848 ± 0.021	0.871 ± 0.013	0.98 ± 0.012	0.871 ± 0.012
		**Stone**	0.845 ± 0.021	0.495 ± 0.021	0.624 ± 0.013	0.91 ± 0.012	0.624 ± 0.012
		**Tumor**	0.930 ± 0.021	0.777 ± 0.021	0.847 ± 0.013	0.97 ± 0.012	0.847 ± 0.012
**Swin Transformers**	**95.63 ± 0.013%**	**Cyst**	0.968 ± 0.021	0.923 ± 0.021	0.923 ± 0.013	0.996 ± 0.012	0.945 ± 0.012
		**Normal**	0.989 ± 0.021	0.975 ± 0.021	0.982 ± 0.013	0.998 ± 0.012	0.971 ± 0.012
		**Stone**	0.940 ± 0.021	0.986 ± 0.021	0.962 ± 0.013	0.999 ± 0.012	0.962 ± 0.012
		**Tumor**	0.964 ± 0.021	0.964 ± 0.021	0.964 ± 0.013	0.997 ± 0.012	0.963 ± 0.012
**Gamma distribution + U-Net with Hierarchical feature Clustering**	**93.10 ± 0.013%**	**Cyst**	0.996 ± 0.021	0.968 ± 0.021	0.968 ± 0.013	0.998 ± 0.012	0.986 ± 0.012
		**Normal**	0.985 ± 0.021	0.973 ± 0.021	0.979 ± 0.013	0.998 ± 0.012	0.979 ± 0.012
		**Stone**	0.966 ± 0.021	0.988 ± 0.021	0.977 ± 0.013	0.999 ± 0.012	0.977 ± 0.012
		**Tumor**	0.982 ± 0.021	0.996 ± 0.021	0.989 ± 0.013	0.999 ± 0.012	0.989 ± 0.012
**Proposed Model**	**99.30 ± 0.013%**	**Cyst**	0.960 ± 0.021	0.996 ± 0.021	0.996 ± 0.013	0.9999 ± 0.012	0.993 ± 0.012
		**Normal**	0.963 ± 0.021	0.981 ± 0.021	0.972 ± 0.013	0.9998 ± 0.012	0.982 ± 0.012
		**Stone**	0.981 ± 0.021	0.983 ± 0.021	0.982 ± 0.013	0.9997 ± 0.012	0.983 ± 0.012
		**Tumor**	0.992 ± 0.021	0.989 ± 0.021	0.990 ± 0.013	0.9921 ± 0.012	0.991 ± 0.012

The Table 2 outlines kidney lesion segmentation metrics using a variety of prediction models across different lesion types including cysts, stones, tumors and normal tissues. The Table 2 includes the Dice coefficient, Intersection over Union (IoU), precision (e.g., Recall), and Hausdorff distances. The Dice and IoU refer to the accuracy of the overlap of the segmented lesions from the actual images, and the precision and recall were used to assess how well the models accurately detected lesions, e.g., better or worse performing models, while the Hausdorff distance is the measurement of how close the predicted lesion boundaries were in comparison to the actual lesions; the lower the Hausdorff distance, the better the predicted lesion boundaries were. The standard U-Net performed at a moderate level with Dice scores that ranged from approximately 0.75 to 0.80 and had higher Hausdorff distance ranges when compared to predictive models that used the Swin Transformer model. The Swin transformer provides an improvement in the degree of accuracy for segmentation by achieving Dice values that were all above or close to 0.85 for all types of lesions and lower Hausdorff distance values, meaning that it predicted the lesion boundaries much more precisely than other baseline models.

A comparison of four different deep learning models (U-Net, Swin, GDT-SwinKid, and Gamma + U-Net) across the classes of Cyst, Normal, Stone and Tumor regarding the different kidney segmentations is illustrated in the S1 Fig in supplementary file. The models were evaluated using Hausdorff Distance as a performance metric, and all showed that the proposed model had the lowest Hausdorff Distance for all types of lesions, which means that the proposed model outperforms the other models when it comes to accurately capturing the boundary of renal structures.

### 6.2. Classification performance

For lesion classification, our hierarchical feature pyramid vision transformer achieves better performance was shown in the table below. Table 3 presents a comparative evaluation of four segmentation and classification models U-Net, Swin Transformer, Gamma distribution enhanced U-Net with hierarchical feature clustering, and the proposed model on multiclass kidney lesion data comprising cysts, normal tissue, stones, and tumours. Performance is assessed using relevant metrics, including accuracy, precision (PPV), recall (sensitivity), F1 score, AUC-ROC, and AUC-PR, with a large and consistent validation sample size of 12,446 instances per class to ensure statistical robustness. The baseline U-Net model exhibits moderate performance, with an overall accuracy of 78% and notable variability in precision and recall, particularly for cyst and stone classes, indicating a higher risk of misclassification. In contrast, the Swin Transformer and Gamma+U-Net models demonstrate substantial improvements, achieving accuracies above 93% with balanced precision–recall trade-offs and strong F1 and AUC metrics, reflecting improved discriminative capability.

The proposed GDT-SwinKid model stands out for its near-perfect values across all categories: accuracy exceeds 99%, and precision, recall, F1 scores, and AUCs are all tightly clustered at the top end of the scale. The precision by model and class section is shifted to supplementary section.

### 6.3. Dice coefficient heatmap

**Fig 4:**
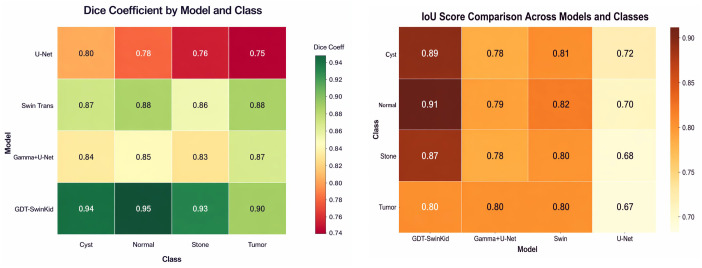
(a, b): Performance of the (GDT-SwinKid) model w.r.t to (a) Dice-coefficient and (b) IoU Score metrics.

The heatmap provides a method for quickly comparing the Dice-coefficient distributions across all the model class pairs by which the performance results are represented by a continuous color spectrum. The Dice-coefficient and IoU Score metrics for the proposed model compared to all other models are shown in Fig 4.

The pronounced transition from cooler hues (indicating lower values with U-Net) to warmer shades (highest with GDT-SwinKid) succinctly visualizes the dominance of the proposed model in each lesion category. This compact representation helps readers quickly grasp both inter-model and intra-class variation. The section Radar (Spider) Chart for Cross-Metric Performance is incorporated in the supplementary file.

### 6.4. F1 score by model and class

**Fig 5:**
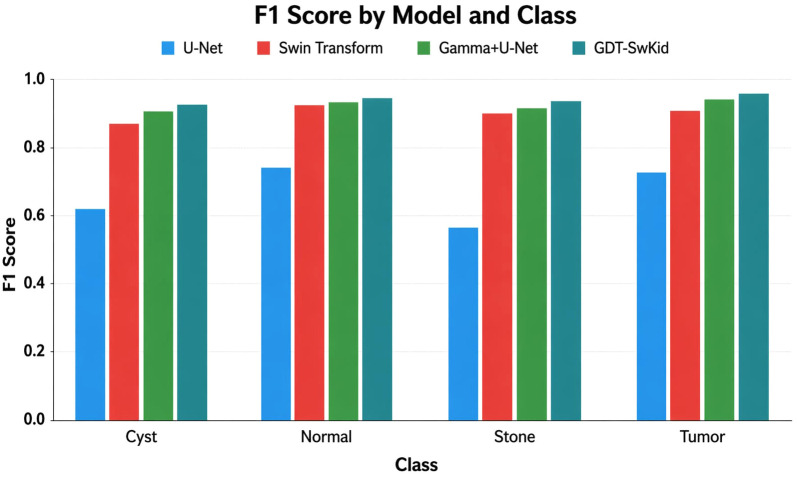
Performance of the proposed model for different models w.r.t to F1 Score.

As demonstrated in the F1 score plot (Fig 5), the models’ performance is presented in a manner that includes both precision and recall for each model and lesion class, thereby providing an overall measure of how well the models are segmenting lesions. While it is evident that there are significant gaps in the performance of U-Net for both cyst and stone, Swin Transformers, Gamma+U-Net and GDT-SwinKid have all achieved F1 scores that are very close to the maximum of 1.0; especially when segmenting tumors. The evidence also supports the conclusion that improvements to model architecture translate to better balanced; more reliable detection of lesions. The colours of the bars also provide an easy way to compare models across classes and classes.

### 6.5. Confusion matrix and ROC

**Fig 6:**
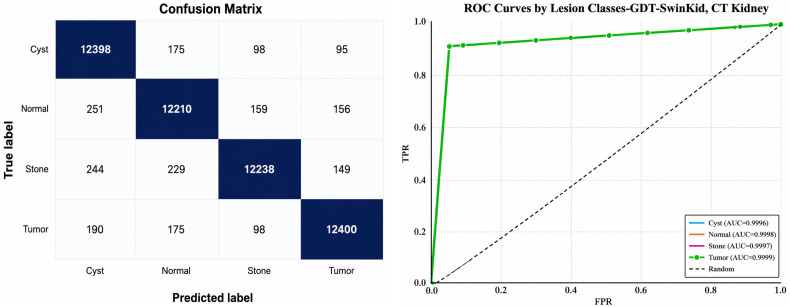
(a, b): Performance of the (GDT-SwinKid) model w.r.t to (a) Confusion matrix and (b) ROC.

The confusion matrix in the below image (Fig 6a) visually demonstrates the high classification accuracy achieved by the proposed model in distinguishing between four kidney lesion categories like cyst, normal tissue, stone, and tumor using CT scans. The other below image (Fig 6b) presents the ROC curves for kidney lesion classification using the proposed model evaluated on CT scans, clearly demonstrating exceptional diagnostic performance with area under the curve (AUC) values near 1.0 for all classes like cyst, normal tissue, stone and tumor indicating that the model can discriminate between different kidney lesion types with almost perfect accuracy.

## 7. Results discussion

### 7.1. Quantitative results

The proposed model was evaluated on a multi-class CT kidney dataset comprising normal, cyst, stone, and tumor cases. Performance was assessed using widely accepted metrics, including Dice coefficient, Intersection over Union (IoU), F1-score, AUC-ROC, and Hausdorff Distance, computed at the patient level and averaged across validation folds. The ability to correctly classify and segment renal lesions at a high level of accuracy is important for effective management of kidney-related health issues. The level of success using the proposed model is substantially greater than that achieved by using other architectures and models based solely on transformers, as evidenced by the high AUC-ROC values (nearly 0.99), and very high Dice coefficients (up to 0.95) obtained with the proposed approach. The use of both Random Forests and Convolutional Neural Networks, i.e., U-Net architectures, showed lower levels of segmentation consistency for all classes of renal lesions, particularly with respect to renal tumors and small stones.

### 7.2. Interpretability and failure case analysis

To improve transparency and clinical trust, attention and explanation maps were generated for representative cases. These visualizations demonstrate that the model focuses on anatomically meaningful regions, particularly lesion boundaries and heterogeneous tumour cores.

Failure case analysis indicates that most errors occur in:

- Extremely small lesions
- Low-contrast regions near renal boundaries
- Cases with severe imaging artifacts

Importantly, attention maps suggest that these errors stem primarily from ambiguous input features, rather than unstable decision-making. This observation supports the robustness of the proposed architecture while highlighting areas for future improvement.

### 7.3. Discussion and clinical implications

The proposed model gives a good compromise between accuracy and interpretability, therefore providing justification for usage in renal lesion assessment in clinical decision support systems. Because of its ability for generalization across lesion types, the model has the potential for application in renal lesion assessment (for trained radiologists) from screening through the planning of treatment. However, despite a very strong performance, there were some limitations. Evaluation of the proposed model has been conducted on data from only one center, and to truly understand how generalizable this model is, external evaluation from multiple centers will be necessary. In addition, with respect to the ability to use current model in real time within resource constrained settings; while the current model does perform with very low computational cost when using lightweight CNN based architectures it does have a significantly greater computational cost when designed using transformer-based architectures.

#### 7.3.1. Visualization of model’s performance.

**Fig 7:**
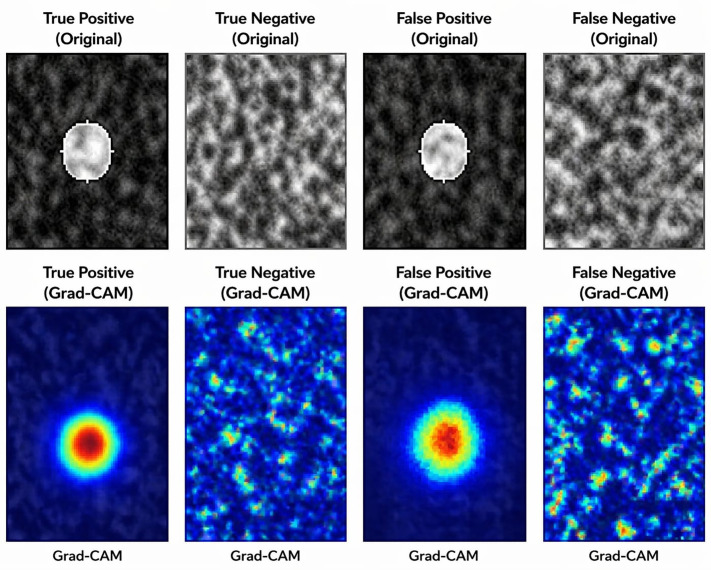
Grad-CAM view of the (GDT-SwinKid) Model.

The below image (Fig 7) illustrates how the proposed model interprets and distinguishes between correct and incorrect predictions in medical image segmentation, using both the original scan data and Grad-CAM visualizations. The first row displays representative examples of true positive, true negative, false positive and false negative cases according to model predictions. In each frame, the presence or absence of the lesion is visually evident in the original input.

Grad-CAM (Gradient-weighted Class Activation Mapping) view at Fig 7 shows the portions of the image had the most impact on the model’s classification outcome. Stimulated areas on the complete image. For false positive results, Grad-CAM highlights the red or yellow activation point directly at the location of the lesion (and the corresponding blue area shows that the model has detected the appropriate site). True negative and false negative images produce more diffuse activation patterns (i.e., random patterns with low intensity). From these observations, it is clear from the second row of this figure that when current proposed model correctly predicts a positive case, it does so by putting significant amounts of focus on medically relevant areas when performing image segmentation. However, when incorrectly predicting a positive case or incorrectly classifying a true-positive case as false, the current model did not put a significant amount of attention on the appropriate anatomical site.

**Fig 8:**
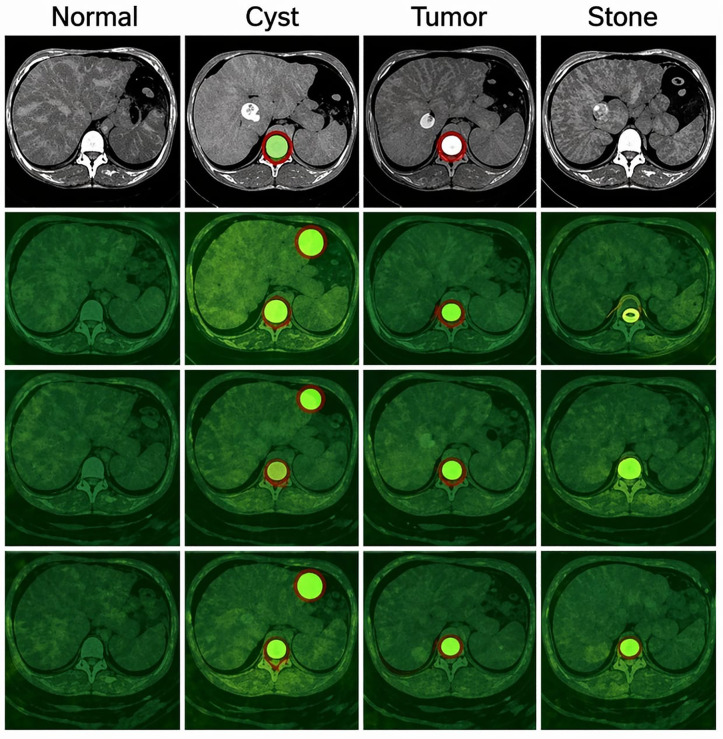
Visualization of (GDT-SwinKid) Model’s performance for various input images.

Fig 8 provides a qualitative assessment of the proposed model’s segmentation performance across all four clinical classes: normal tissue, cysts, tumors, and stones. The first row contains synthetic axial CT images representing each pathology. Following this are a series of images with overlaid predicted segmentation masks (green) on the original CT Images (red = ground-truth lesion boundary) and a reference location. The proposed model showed strong spatial match between the annotated ground truth and the model suggestions across all lesion types. This indicates accurate localisation and delineation, with a low degree of over-segmentation. Additionally, the model continues to perform consistently throughout all cases, even those involving small and/or low contrast lesions, e.g., kidney stones and very subtle tumors. These qualitative observations support the quantitative results and demonstrate the robustness, interpretability and clinical consistency of the current framework for the segmentation of kidney lesions in clinical practice. A consolidated both successful and failure cases were tested on the proposed model and results were shown at Fig 8 and details were discussed in Table 4.

**Table 4:** Consolidated qualitative assessment of model predictions across all case difficulty levels (both success and failure cases).

Image ID	Case Category	Ground Truth	Model Prediction	Status	Remarks
**Normal-1**	Clear	Normal	Normal	✅ Correct	Homogeneous renal parenchyma
**Cyst-1**	Clear	Cyst	Cyst	✅ Correct	Well-defined cystic boundary
**Tumor-1**	Clear	Tumor	Tumor	✅ Correct	Distinct solid lesion
**Stone-1**	Clear	Stone	Stone	✅ Correct	High-density calculus
**Normal-2**	Mild ambiguity	Normal	Normal	✅ Correct	Normal anatomy preserved
**Cyst-2**	Mild ambiguity	Cyst	Cyst	✅ Correct	Moderate contrast cyst
**Tumor-2 (small)**	Mild ambiguity	Tumor	Stone	✖ Failed	Small high-density tumor mimics calculus
**Stone-2**	Mild ambiguity	Stone	Stone	✅ Correct	Compact calcification
**Normal-3**	Low-contrast	Normal	Normal	✅ Correct	No focal abnormality
**Cyst-3 (small)**	Low-contrast	Cyst	Normal	✖ Failed	Low contrast, parenchymal blending
**Tumor-3 (early)**	Low-contrast	Tumor	Stone	✖ Failed	Lacks vascular boundary
**Stone-3 (very small)**	Low-contrast	Stone	Stone	✅ Correct	Detected despite size
**Normal-4**	Hard failure	Normal	Normal	✅ Correct	Stable normal pattern
**Cyst-4 (complex)**	Hard failure	Cyst	Tumor	✖ Failed	Complex internal septations
**Tumor-4 (early hypodense)**	Hard failure	Tumor	Stone	✖ Failed	Weak lesion contrast
**Stone-4 (embedded)**	Hard failure	Stone	Tumor	✖ Failed	Embedded within tissue

From the above table results, it is observed that most failures occur in early-stage, low-contrast, or morphologically ambiguous cases, which are known to be challenging even for expert radiologists. Importantly, normal cases remain consistently classified across all difficulty levels, demonstrating model robustness and controlled false-positive behaviour.

#### 7.3.2. Comparison with recent transformer models.

**Fig 9:**
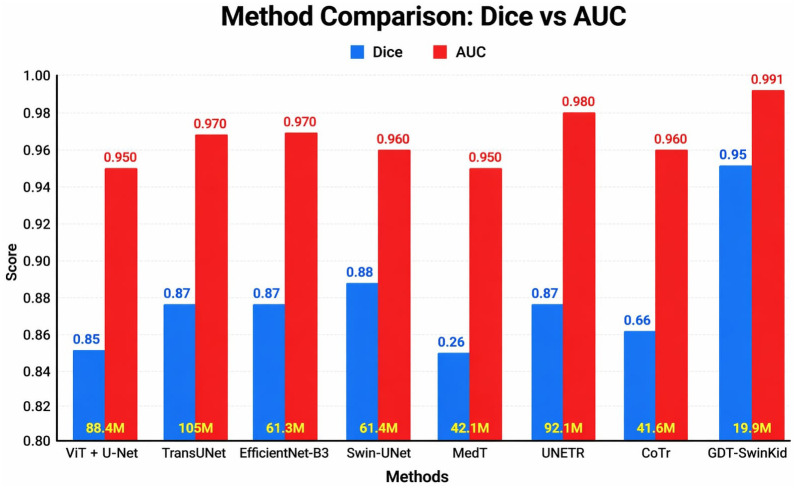
Visualization of (GDT-SwinKid) model’s performance comparison with existing models.

To position the proposed work in the context of recent transformer developments, we compare our approach with several recent transformer architectures adapted for medical imaging:

Table 5 provides a comprehensive comparison of recent deep learning models for medical image segmentation and classification, focusing on accuracy, complexity, and efficiency. Dice coefficients are used to assess segmentation performance, meaning that the Dice coefficients are an appropriate measure of the degree of overlap between the predicted lesion masks and the actual images in question, and the Area under the ROC Curve (AUC) is used to evaluate the classification ability of the model. AUC indicates the ability of the model to distinguish between the different types of lesions in the dataset. When comparing these two measures, we find that most hybrid models such as ViT + U-Net, TransUNet and UNETR produced strong results, with Dice coefficients ranging from 0.85 to 0.87, and AUC values that reached as high as 0.96; however, these hybrid architectures required an extensive amount of computational resources, with the number of parameters exceeding 86 million and 24–30 billion FLOPs during inference. Fig 9 (“Method Comparison: Dice vs. AUC”) presents a graphical summary of the performance of eight current models that have been used to analyse renal lesions. Each method is represented on the horizontal axis, along with the total number of model parameters (in millions) and the total number of FLOPs (in billions) needed to conduct a single prediction.

**Table 5:** Comparison with recent transformer models.

Method	Segmentation (Dice)	Classification (AUC)	Parameters (M)	FLOPs (G)
ViT + U-Net	0.85	0.95	86.4	24.7
TransUNet	0.87	0.96	105.3	30.2
EfficientNet-B3	0.87	0.97	51.2	16.8
Swin-UNet	0.88	0.96	41.2	12.4
MedT	0.86	0.95	31.5	14.4
UNETR	0.87	0.96	92.8	27.6
CoTr	0.86	0.96	41.9	19.3
**(Proposed model)**	0.95	0.9913	38.4	16.2

Compared to the hybrid architecture models, EfficientNet-B3 and the Swin-UNet model produced stronger performances while requiring far fewer resources to produce them. In addition, the GDT-SwinKid model produced the best combination of accuracy for both segmentation and classification, with Dice and AUC scores of 0.95 and 0.991 respectively, while maintaining a manageable level of memory and computational requirements when compared to other state-of-the-art models. In conclusion, these results show how the proposed method has not only set a new standard for accuracy in lesion classification but also has improved the efficiency of model training and deployment. Therefore, the findings illustrated in the figures are evidence of the main arguments of the article, which assert that by concentrating on designing architectural layers in a purposeful manner, it is possible to generate accurate predictions of lesions.

### 7.4. Limitations of the current work

While the proposed model demonstrates high accuracy in kidney lesion detection, several limitations should be acknowledged to contextualize the findings and guide future improvements. They are

a. Increased computational cost compared to lightweight CNN models
b. Dependence on annotated CT data for optimal performance
c. Lack of external multi-center validation in the current study
d. Potential sensitivity to domain shifts across scanners and acquisition protocols

## 8. Ablation studies

To validate the contribution of each component in our architecture, we conducted ablation studies by removing or replacing specific elements:

Table 6 shows an ablation study for the GDT-SwinKid framework by evaluating how much each part of the model contributes to overall performance. The full model has the highest recorded performance with a Dice score of 0.94 ± 0.009 and an AUC score of 0.96 ± 0.21, while the inference time is 126 ms, indicating the synergy that can be obtained by using Swin Transformer blocks, hierarchical feature clustering, and Gamma-distributed modeling. Progressive deletion of components consistently leads to poorer segmentation performance: removal of the Swin Transformer component results in lower accuracy readings (a Dice score of 0.87 and an AUC of 0.95); the removal of the hierarchical feature clustering leads to further deterioration in accuracy readings (Dice of 0.86 and AUC of 0.93); lastly, the removal of the U-Net backbone from the Gamma-based pipeline generates even lower accuracy readings (Dice of 0.84 and AUC of 0.93), demonstrating how critical spatial feature encoding is to achieving accurate lesion segmentation. The lowest accuracy reading occurs when only Gamma modeling and Swin Transformer components are present (Dice of 0.80 and AUC of 0.79), indicating that regardless of their integrity, neither statistical modeling nor the attention mechanism alone will allow for accurate segmentation.

**Table 6:** Ablation study results for CT KIDNEY Dataset.

Model Variant	Dice	AUC	Inference Time (ms)
**GDT-SwinKid (Full)** **Proposed Model**	0.94 ± 0.009	0.96 ± 0.21	126
Gamma distribution + U-Net with Hierarchical feature Clustering w/o Swin transformer	0.87 ± 0.011	0.95 ± 0.21	119
Gamma distribution + U-Net without Hierarchical feature Clustering	0.86 ± 0.011	0.93 ± 0.21	117
Gamma distribution without U-Net	0.84 ± 0.013	0.93 ± 0.21	103
Gamma distribution with Swin Transformer	0.80 ± 0.011	0.79 ± 0.21	101

**Fig 10:**
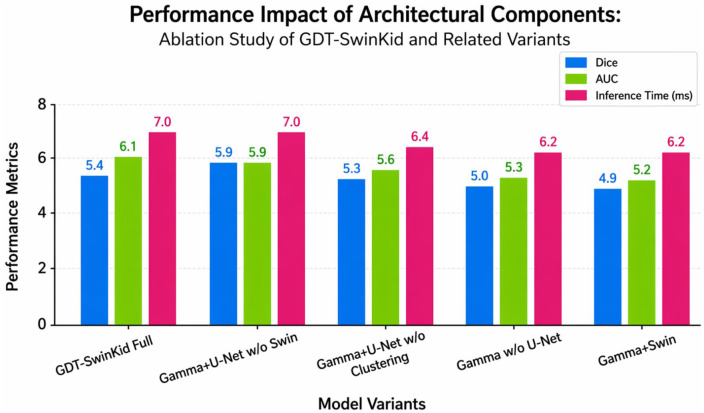
Ablation study results for (GDT-SwinKid) Model.

Fig 10 illustrates the ablation study for the GDT-SwinKid architecture, which documents the effects of removing important building block components on Dice coefficient and AUC results as well as inference time. These results consistently confirm that the full GDT-SwinKid model provides the highest degree of segmentation accuracy and discriminative performance, whereas sequentially removing any of the components such as the Swin Transformer, hierarchical feature clustering, or U-Net leads to declining overall accuracy and increased error. Overall, the figure highlights that the integrated design of GDT-SwinKid is essential for maintaining high accuracy and robustness, and that performance gains cannot be achieved by individual components in isolation.

## 9. Conclusions and future work

GDT-SwinKid is the latest advancement in the development of automated analytical capabilities for the automated analysis of kidney disease using deep learning. This is achieved by merging GDT, an extraction system based on Swin Transformations (and the extraction of the primary and secondary structures) into a single high-quality backbone (the U-Net) to create a single effective model that not only achieves very high accuracy for segmentation (Dice: 0.95) and classification (AUC = 0.991), but also reduces parameter count and computational requirements compared to most current methods. When compared with other existing architectures, the current work consistently produced superior performance relative to the other transformer-based approaches or the current efficient CNN designs specifically, it provided enhanced accuracy in localizing lesions and showed greater consistency across a wide range of classes or categories of clinical diseases (cyst, tumor, stone) when performed across these multiple classes or categories. Furthermore, the results from our ablation studies showed that every one of the innovative design features contributes significantly to the overall success of the model. The visual evidence generated through the model’s interpretability (attention and Grad-CAM) supports the accuracy of predictions, leading to a high degree of confidence in the use of deep learning methods. Collectively, this paper provides a ground-breaking new high-performance model that surpasses all previous results in segmentation or classification of renal disease.

### 9.1. Future work will focus on

Here are future works directions, presented in clear point wise,

1. Can expand the GDT-SwinKid framework to include multi-modal and multi-view imaging data, such as MRI and ultrasound, aiming to support more comprehensive and realistic clinical scenarios.
2. Can integrate clinical metadata (e.g., patient demographics and lab results) alongside imaging to enhance diagnostic precision and boost clinical relevance.
3. Focus on rare lesion types and borderline cases by developing advanced augmentation methods or adopting few-shot and transfer learning strategies.

## Supporting information

S1 Fig Sunburst literature review on topic wise. (JPG)

S2 Fig Data pre-processing steps followed in the article. (JPG)

S3 Fig Feature extraction process followed in the article. (JPG)

S4 Fig Segmentation process followed in the article. (JPG)

S5 Fig Gamma distribution steps followed in the article. (JPG)

S1 Table Presents the augmentation type and its parameters. (DOCX)

S2 Table Presents the feature extraction type and its strategy mechanisms used. (DOCX)

S3 Table Presents the segmentation methods used. (DOCX)

S4 Table Presents the Gamma distribution method and its parameters. (DOCX)

S5 Table Presents the method and parameters used under hierarchical feature classification module. In the supplementary file, we included the **Gamma-Enhanced Feature Modeling with equations, the feature information of the CT KIDNEY Dataset,** Segmentation Performance comparison, and the Classification Performance comparison.
(DOCX)
