# Supplementary figures and images for: GDT-SwinKid: A hybrid model for precise renal lesion analysis
Source: PLoS One. 2026 May 20;21(5):e0349285. doi: 10.1371/journal.pone.0349285 (PMC13189418; doi:10.1371/journal.pone.0349285)

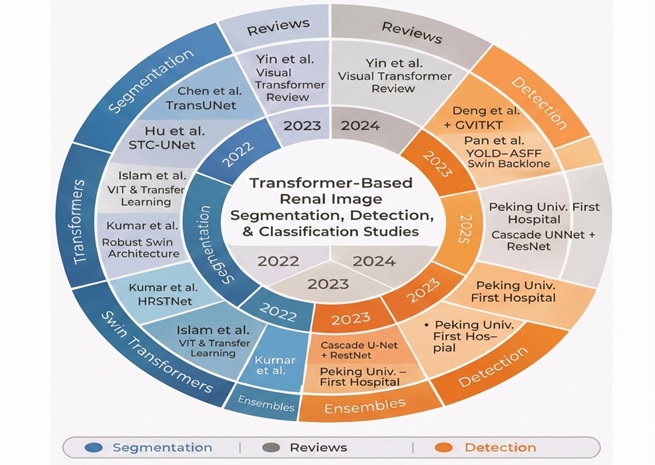

Supplement: S1 Fig — (JPG) [file pone.0349285.s001.jpg]

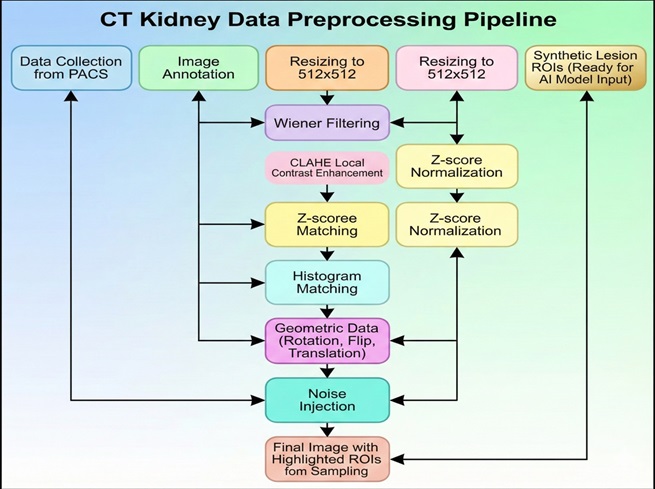

Supplement: S2 Fig — (JPG) [file pone.0349285.s002.jpg]

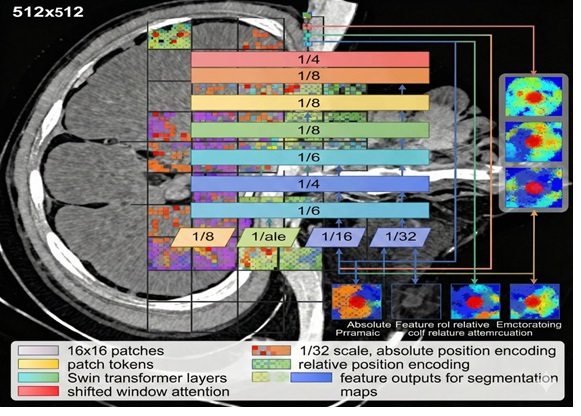

Supplement: S3 Fig — (JPG) [file pone.0349285.s003.jpg]

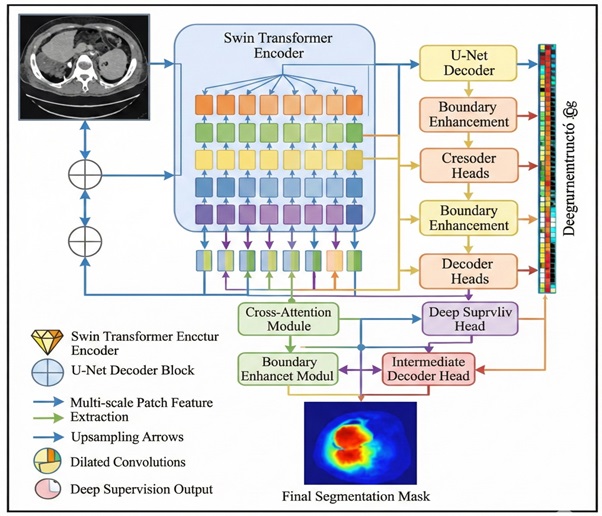

Supplement: S4 Fig — (JPG) [file pone.0349285.s004.jpg]

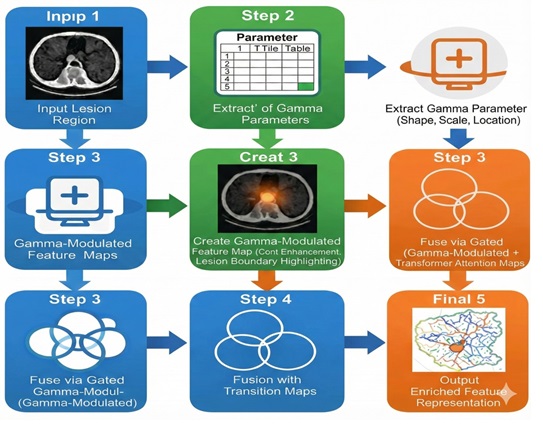

Supplement: S5 Fig — (JPG) [file pone.0349285.s005.jpg]
