# Supplementary material for: GDT-SwinKid: A hybrid model for precise renal lesion analysis
Source: PLoS One. 2026 May 20;21(5):e0349285. doi: 10.1371/journal.pone.0349285 (PMC13189418; doi:10.1371/journal.pone.0349285)
Supplement: S1 Table — (DOCX) [file pone.0349285.s006.docx]

**Table S1:** Presents the augmentation type and its parameters

| **Augmentation Type** | **Parameters** | **Objective** |
| --- | --- | --- |
| Geometric | Rotate ±15°, flip, translate ±10%, scale 0.9–1.1 | Model generalization |
| Intensity | Brightness/contrast ±10%, Gamma 0.8–1.2 | Simulate clinical variation |
| Noise/Blur | Gaussian σ=0.01, blur σ=0.5 | Mimic artifacts, add robustness |
| Lesion-aware | Oversample/transform minority/difficult lesions | Boost rare/small lesion detection |
