# Supplementary material for: GDT-SwinKid: A hybrid model for precise renal lesion analysis
Source: PLoS One. 2026 May 20;21(5):e0349285. doi: 10.1371/journal.pone.0349285 (PMC13189418; doi:10.1371/journal.pone.0349285)
Supplement: S2 Table — (DOCX) [file pone.0349285.s007.docx]

**Table S2:** Presents the feature extraction type and its strategy mechanisms used

| **Step** | **Transformer Strategy / Mechanism** | **Purpose** |
| --- | --- | --- |
| Patch Embedding | 16×16 non-overlapping patches, linear/convolutional map | Tokenize images for transformer input |
| Hierarchical Learning | Swin Transformer, shifted windows, patch merging | Capture multi-scale local/global context efficiently |
| Multi-scale Pyramid | Extract at 1/4, 1/8, 1/16, 1/32 resolutions | Combine features for detail and structure |
| Provide spatial awareness | Provide spatial awareness | Provide spatial awareness |
| Self-attention Maps | Save maps from intermediate layers | Guide segmentation, enable interpretability |
