# Supplementary material for: GDT-SwinKid: A hybrid model for precise renal lesion analysis
Source: PLoS One. 2026 May 20;21(5):e0349285. doi: 10.1371/journal.pone.0349285 (PMC13189418; doi:10.1371/journal.pone.0349285)
Supplement: S3 Table — (DOCX) [file pone.0349285.s008.docx]

**Table S3:** Presents the segmentation methods used

| **Step** | **Method** | **Purpose** |
| --- | --- | --- |
| Encoder | Swin Transformer blocks, hierarchical feature multi-scale maps | Rich feature extraction |
| Decoder | U-Net upsampling with skip connections | Recover spatial precision |
| Boundary Enhancement | Dedicated module, dilated convolutions | Improve boundary localization |
| Deep Supervision | Auxiliary segmentation heads at multiple levels | Stronger, multi-scale feedback |
