# Supplementary material for: GDT-SwinKid: A hybrid model for precise renal lesion analysis
Source: PLoS One. 2026 May 20;21(5):e0349285. doi: 10.1371/journal.pone.0349285 (PMC13189418; doi:10.1371/journal.pone.0349285)
Supplement: S4 Table — (DOCX) [file pone.0349285.s009.docx]

**Table S4:** Presents the Gamma distribution method and its parameters

| **Step** | **Method/Mechanism** | **Objective** |
| --- | --- | --- |
| Parameter Estimation | Fit α, β, μ of Gamma distribution | Capture statistical intensity properties |
| Feature Enhancement | Gamma-modulated feature map | Enhance abnormal/normal contrast |
| Fusion with Transformers | Adaptive gating with attention maps | Integrate local stats with global structure cues |
