# Supplementary material for: GDT-SwinKid: A hybrid model for precise renal lesion analysis
Source: PLoS One. 2026 May 20;21(5):e0349285. doi: 10.1371/journal.pone.0349285 (PMC13189418; doi:10.1371/journal.pone.0349285)
Supplement: S5 Table — In the supplementary file, we included the Gamma-Enhanced Feature Modeling with equations, the feature information of the CT KIDNEY Dataset, Segmentation Performance comparison, and the Classification Performance comparison. (DOCX) [file pone.0349285.s010.docx]

**Table S5:** Presents the method and parameters used under hierarchical feature classification module

| **Step** | **Method/Mechanism** | **Purpose** |
| --- | --- | --- |
| Input Preparation | Resize cropped lesion to 224×224 | Standardized input size |
| Feature Extraction | Pyramid Vision Transformer (multi-scale) | Capture of hierarchical lesion features |
| Global Context Modeling | Class token aggregation | Robust global diagnosis |
| Feature Fusion | Attention-based multi-scale fusion | Integrate detailed and coarse cues |
| Classification Head | MLP with dropout | Probability output, regularization |
